# Effects of Highly Oxygenated Water in a Hyperuricemia Rat Model

**DOI:** 10.1155/2020/1323270

**Published:** 2020-01-30

**Authors:** Chih-Hsiang Fang, Cheng-Chia Tsai, Yan-Jye Shyong, Chun-Ting Yang, Keng-Yuan Li, Yi-Wen Lin, Kuo-Chi Chang, Mao-Hsien Wang, Tang-Ming Wu, Feng-Huei Lin

**Affiliations:** ^1^Institute of Biomedical Engineering, National Taiwan University, No. 1, Sec. 4, Roosevelt Rd., Taipei 10617, Taiwan; ^2^Department of Neurosurgery, Mackay Memorial Hospital, No. 92, Sec. 2, Zhongshan N. Rd., Taipei 10449, Taiwan; ^3^Graduate Institute of Injury Prevention and Control, Taipei Medical University, 250 Wuxing St., Taipei 11031, Taipei, Taiwan; ^4^School of Pharmacy and Institute of Clinical Pharmacy and Pharmaceutical Sciences, National Cheng Kung University, No. 1, University Road, Tainan 701, Taiwan; ^5^Department of Chemical Engineering and Biotechnology, National Taipei University of Technology, No. 1, Sec. 3, Zhongxiao E. Rd., Taipei 10608, Taiwan; ^6^Department of Anesthesia, En Chu Kong Hospital, No. 399, Fuxing Rd., Sanxia District, Taipei 237, Taiwan; ^7^Division of Medical Engineering, National Health Research Institute, 35 Keyan Road, Zhunan, Miaoli County 35053, Taiwan

## Abstract

Recent years have seen a rapidly rising number of oxygenated water brands that claim to impart health benefits and increase athletic performance by improving oxygen availability in the body. Drinks with higher dissolved oxygen concentrations have in recent times gained popularity as potential ergogenic aids, despite the lack of evidence regarding their efficacy. The aim of this study was to characterize oxygenated water and assess the improvement in uric acid metabolism while identifying performance enhancements in animals administered oxygenated water. Oxygenated water was characterized by hydrogen and oxygen nuclear magnetic resonance (NMR) spectroscopy. Hyperuricemia in rats was induced by treatment with oxonic acid potassium salt, and the animals were given oxygenated drinking water before, during, or after oxonic acid treatment. Serum uric acid was measured to confirm the effects on uric acid metabolism. Following oxygenation, the full width at half maximum (FWHM) was reduced to 11.56 Hz and 64.16 Hz in the hydrogen and oxygen NMR spectra, respectively. Oxygenated water molecule clusters were reduced in size due to the reduction in FWHM. Oxygen concentration did not vary significantly with increased temperature. However, standing time played a critical role in the amount of oxygen dissolved in the water. The rat studies indicated that oxygenated water reduced serum uric acid levels and their rate of increase and enhanced uric acid metabolism. A significant improvement in uric acid metabolism and rate of increase in serum uric acid concentration was observed in hyperuricemic rats administered oxygenated water compared to that in rats administered regular water. High oxygen concentrations enhanced the rate of oxygen absorption, leading to increased glycolysis and mitochondrial protein synthesis. Therefore, oxygenated water is a potential adjuvant therapy or health food for treatment of hyperuricemia.

## 1. Introduction

Uric acid is the final product of purine metabolism in most mammals and is degraded to allantoin by the hepatic enzyme uricase; elevated serum uric acid (hyperuricemia) is a well-known risk factor for gout [1]. Hyperuricemia is associated with an increased risk of development of hypertension and it additionally confers increased risk of cardiovascular death. Several drugs, such as nonsteroidal anti-inflammatory drugs (NSAIDs), colchicine, and benzbromarone, are currently available for the treatment of gout. In addition to drug treatment, diet and drinking water are other ways to control the serum concentration of uric acid, as shown in Table 1.

Since the early 1990s, several manufacturers, particularly in Europe and the United States, have produced drinking water with increased concentrations of dissolved oxygen, ranging from 30 to 120 mg/L water. Regular drinking water contains 5–7 mg of dissolved oxygen per liter and fresh fountain water contains 10–12 mg/L. The manufacturers argue that consumption of oxygenated water improves oxygen availability and the “overall metabolism and health as well as the resistance against pollutants and drugs.” However, these statements are controversial since they are based on anecdotal reports, and there is a lack of scientific evidence to corroborate these claims. In the 1970s, several groups studied the effects of insufflation of gaseous oxygen into the intestinal lumen. Gelman et al. showed, both clinically and experimentally, that oxygen application into the intestinal lumen improved hepatic circulation and the overall oxygen supply [2, 3]. Recently, Forth and Adam reported that oxygenated water administered intragastrically to rabbits increased oxygen content within the abdominal cavity and portal vein [4]. Apart from these experimental data, there are some clinical reports that describe positive effects of oxygenated water for patients suffering from a large variety of diseases, such as morbid obesity, cholecystitis, and portal hypertension [5]. In clinical studies, Eble and Wannenmacher studied the oxygenation of lymph node metastases in patients suffering from head and neck carcinomas and demonstrated increased oxygen in the lymph node tissue of patients after drinking oxygenated water (60 mg oxygen/L H_2_O) [6]. In addition, Handajani et al. demonstrated that oxygenated water could reduce postprandial glucose levels in diabetes mellitus (DM) subjects. DM subjects with normal nutritional states also had a greater tendency for malondialdehyde (MDA) reduction after consuming oxygenated water for 45 days. Most subjects felt healthier after consuming oxygenated water [7]. Based on these studies, we believe that oxygenated water might improve cellular metabolism.

Several reports have speculated that oxygenated water provides physiological and immunological benefits for the body. However, to the best of our knowledge, there are no data on the effects of oxygenated water on hyperuricemia. The aim of this study was to estimate the improvement in uric acid metabolism by identifying any performance enhancement in Wistar rats administered oxygenated water.

## 2. Materials and Methods

### 2.1. Characterization of Oxygenated Water

Oxygenated water was characterized by 11.74 Tesla (^1^H) hydrogen and (^17^O) oxygen nuclear magnetic resonance (NMR) spectroscopy. The sample (540 *μ*L) and 60 *μ*L D_2_O were mixed in a 5 mm NMR spectroscopy tube and analyzed under the following conditions: 67.768 MHz resonance frequency, 0.345 s sampling time, 5940.4 Hz bandwidth, 4096 scans, 67° flip angle, 0.2 s relaxation delay, and room temperature (25°C). The reference oxygen concentrations were determined by an electrochemical method using a WTW Oxi3210 with a CellOx 325 electrode (WTW, Weilheim).

### 2.2. Animals and Experimental Design

**Figure 1:**
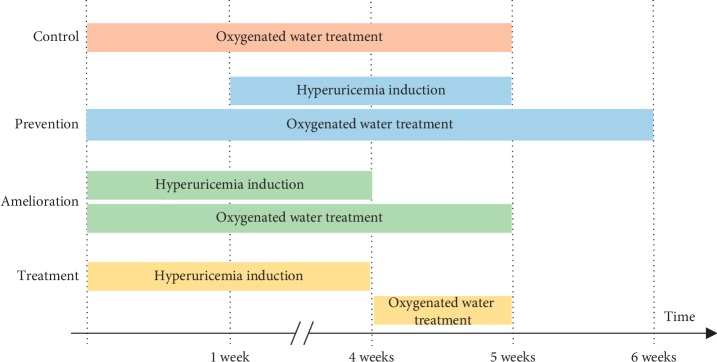
Experimental design in control, prevention, amelioration, and treatment groups.

Male Wistar rats (*n* = 60, as estimated by multiple regression analysis; body weight: 200–250 g; 8 weeks old) (BioLASCO Taiwan Co., Taiwan) were used in all experiments, and serum uric acid was measured at multiple time points. All animal procedures and experiments were performed following a protocol approved by the National Taiwan University College of Medicine and Public Health Institutional Animal Care and Use Committee (IACUC NO. 20130429). Small molecule high oxygenated water (75H30p) was purchased from O_2_xy Young Co., Taiwan. A total of 80 mL drinking water (containing either 0, 10, 50, or 80 mL oxygenated water) was given to each animal per day throughout one of three different time intervals to simulate prevention, amelioration, and treatment of hyperuricemia, as shown in Table 2 and Figure 1. The control group consisted of healthy rats without induction of hyperuricemia (see below). All animals were euthanized by CO_2_ asphyxiation at the end of the study. The CO_2_ was delivered from a compressed gas tank at a flow rate of 10–30% of the cage volume/min. Death was confirmed by examination of animal respiratory arrest and cervical dislocation as a secondary method of euthanasia.

### 2.3. Induction of Hyperuricemia

In a previous study, Mazzali et al. [8] compared serum uric acid levels in animal models of hyperuricemia induced by oxonic acid potassium salt (OA) with and without uric acid supplements, by renal histology. The authors reported that rats treated with 2% (w/v) OA could develop hyperuricemia, with a well-preserved renal architecture and no intrarenal urate crystals. Therefore, we followed this previous protocol to induce hyperuricemia in our study. OA powder was mixed with sterile distilled water to prepare 2% (w/v) OA solution, which was administered at 4 mL every 3 days for 4 weeks by intraperitoneal injection (IP) to induce hyperuricemia. Serum uric acid was measured at multiple time points to confirm induction of hyperuricemia.

### 2.4. Prevention of Hyperuricemia

In the prevention of hyperuricemia experiment, all animals (prevention group, *n* = 10; control group, *n* = 10) were pretreated with oxygenated water for one week before hyperuricemia induction. After pretreatment, hyperuricemia was induced in the animals by OA for 4 weeks, followed by continued treatment with oxygenated water for one week. After the 6-week experiment, serum uric acid was analyzed (*n* = 10 rats per group). The experimental design is shown in Table 2 and Figure 1.

### 2.5. Amelioration of Hyperuricemia

In the amelioration of hyperuricemia experiment, oxygenated water treatment and induction of hyperuricemia were initiated simultaneously. All animals (amelioration group, *n* = 10; control group, *n* = 10) were treated with oxygenated water and induced by OA for 4 weeks. After the 4-week induction, the oxygenated water treatment was continued for an additional week. After the 5-week experiment, serum uric acid was analyzed (*n* = 10 rats per group). The experimental design is shown in Table 2 and Figure 1.

### 2.6. Treatment of Hyperuricemia

In the hyperuricemia treatment experiment, all animals (treatment group, *n* = 10; control group, *n* = 10) were treated with OA for 4 weeks to induce hyperuricemia, followed by oxygenated water treatment for one week. After the 5-week experiment, serum uric acid analysis was performed (*n* = 10 rats per group). The experimental design is shown in Table 2 and Figure 1.

### 2.7. Serum Uric Acid Analysis

Once a week, a total of 0.5 mL blood was sampled from the lateral tail vein, using a 26 G needle (TERUMO, Japan), a one-mL syringe (TERUMO, Japan), and blood collection tubes (Vacutainer® PST™, Becton, Dickinson, and Company, USA). Serum specimens were collected after centrifugation (MC-CUBEE, AAT Bioquest, Canada) at 3000 ×g for 10 min. Serum uric acid was measured by the National Taiwan University Veterinary Hospital, Taiwan.

### 2.8. Statistics

Values and concentrations are expressed as means ± SE. Statistical significance (*p* > 0.05) of serum uric acid levels between the groups was evaluated by unpaired Student's *t*-tests or ANOVA with Fisher's protected least significant difference test for multiple comparisons.

## 3. Results and Discussion

### 3.1. Characterization of Oxygenated Water

**Figure 2:**
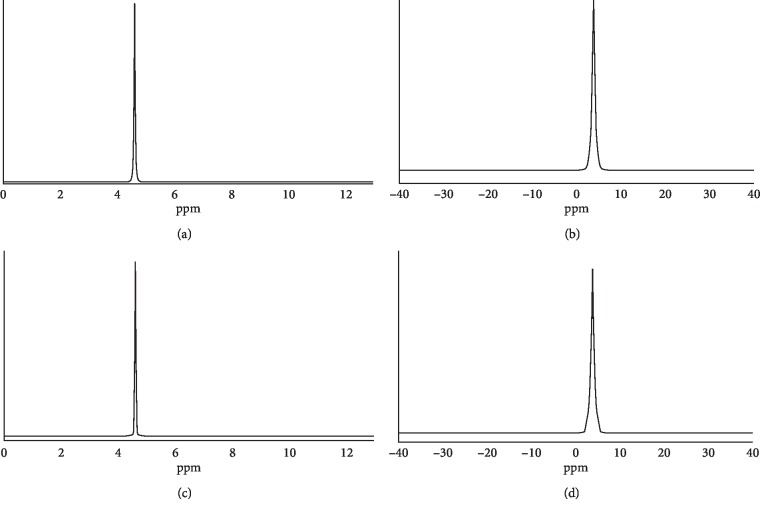
NMR analysis of oxygenated water. (a) ^1^H NMR spectroscopy of unoxygenated water, (b) ^17^O NMR spectroscopy of unoxygenated water, (c) ^1^H NMR spectroscopy of oxygenated water, and (d) ^17^O NMR spectroscopy of oxygenated water.

We used (^1^H) hydrogen and (^17^O) oxygen NMR spectroscopy to measure the full width at half maximum (FWHM) for oxygenated water. As shown in Figure 2, the FWHM of unoxygenated water was 13.34 Hz and 107.65 Hz with hydrogen and oxygen NMR spectroscopy, respectively. Following the oxygenation process, the FWHM was reduced to 11.56 Hz and 64.16 Hz, respectively. The oxygen concentration of unoxygenated water was 3.7 mg/L, and after 3.5 h of oxygenation, the oxygen concentration rose to 38.5 mg/L. Subsequently, oxygen precipitation in the oxygenated water was measured at different temperatures and standing times as indicated in Tables 3 and 4, respectively. We did not observe a significant variation in oxygen concentration (37 mg/L to 32 mg/L) when the temperature was increased from 10 to 50°C. However, standing time played a more critical role in the amount of oxygen dissolved in the water. As indicated in Table 4, the oxygen concentration decreased from 29.2 mg/L to 16.8 mg/L when the standing time was 24 h.

### 3.2. Serum Uric Acid Analysis in Prevention of Hyperuricemia

**Figure 3:**
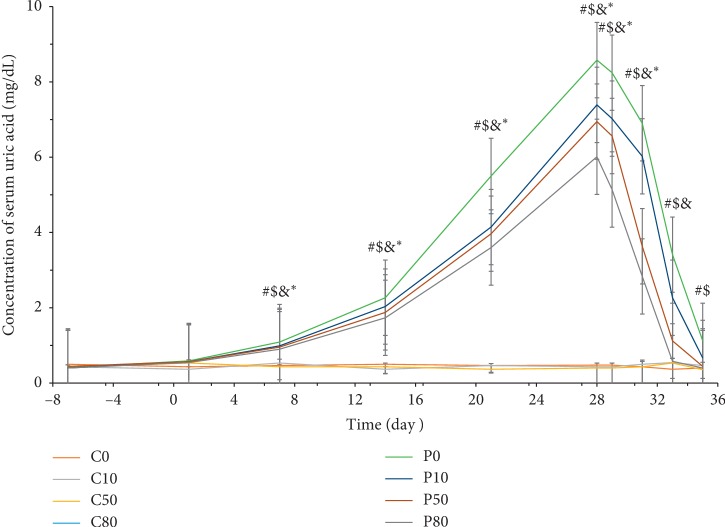
The concentration of serum uric acid changes in the prevention of hyperuricemia during OA induction and oxygenated water treatment. Data are expressed as means ± S.E.M. (*n* = 10) (^#^*p* value <0.05 of P0 compared to C0; ^$^*p* < 0.05 of P10 compared to C10; ^&^*p* < 0.05 of P50 compared to C50; ^*∗*^*p* < 0.05 of P80 compared to C80).

In the experiments on the prevention of hyperuricemia, when drinking regular water, serum uric acid concentrations were observed to increase from 0.41 mg/dL to 8.58 mg/dL in P0, from 0.40 mg/dL to 7.39 mg/dL in P10, from 0.44 mg/dL to 6.94 mg/dL in P50, and from 0.40 mg/dL to 6.01 mg/dL in P80 (Figure 3). There was no significant difference in the control groups (C0, C10, C50, and C80), as the concentrations of serum uric acid in the control groups were 0.45 mg/dL to 0.50 mg/dL. Furthermore, serum uric acid concentration was progressively attenuated in hyperuricemic rats with increased consumption of oxygenated water, when compared to that with consumption of normal drinking water. After oxygenated water treatment, the concentration of serum uric acid decreased to 1.14 mg/dL in P0, 0.84 mg/dL in P10, 0.49 mg/dL in P50, and 0.43 mg/dL in P80.

### 3.3. Serum Uric Acid Analysis in Amelioration of Hyperuricemia

**Figure 4:**
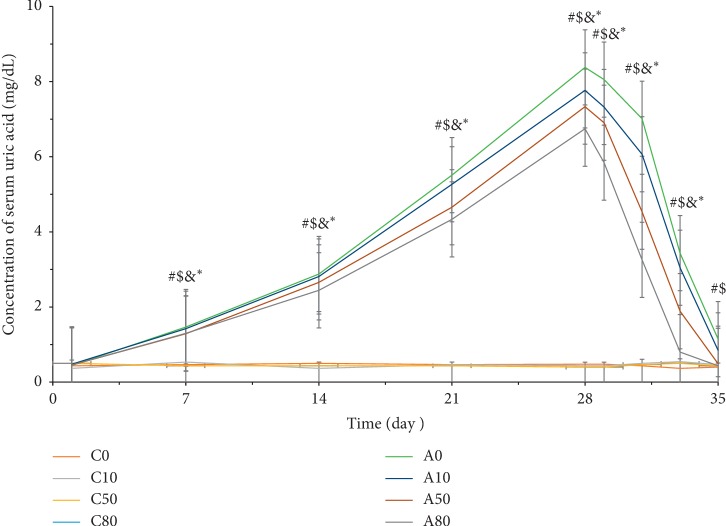
The concentration of serum uric acid changes in amelioration of hyperuricemia during OA induction and oxygenated water treatment. Data are expressed as means ± S.E.M. (*n* = 10) (^#^*p* value <0.05 of A0 compared to C0; ^$^*p* < 0.05 of A10 compared to C10; ^&^*p* < 0.05 of A50 compared to C50; ^*∗*^*p* < 0.05 of A80 compared to C80).

As shown in Figure 4, the concentration of serum uric acid increased from 0.44 mg/dL to 8.38 mg/dL in A0, from 0.48 mg/dL to 7.77 mg/dL in A10, from 0.46 mg/dL to 7.33 mg/dL in A50, and from 0.46 mg/dL to 6.74 mg/dL in A80 after a 4-week induction of hyperuricemia. Similar to the results for the prevention of hyperuricemia, there was no significant difference in control group values (C0, C10, C50, and C80), as the concentrations of serum uric acid in the control groups were 0.45 mg/dL to 0.50 mg/dL. Serum uric acid concentration was significantly lowered in hyperuricemic animals with increased consumption of oxygenated water, when compared to animals drinking normal water. Furthermore, the concentration of serum uric acid decreased to 1.12 mg/dL in A0, 0.67 mg/dL in A10, 0.44 mg/dL in A50, and 0.40 mg/dL in A80 after oxygenated water treatment.

### 3.4. Serum Uric Acid Analysis in Treatment of Hyperuricemia

**Figure 5:**
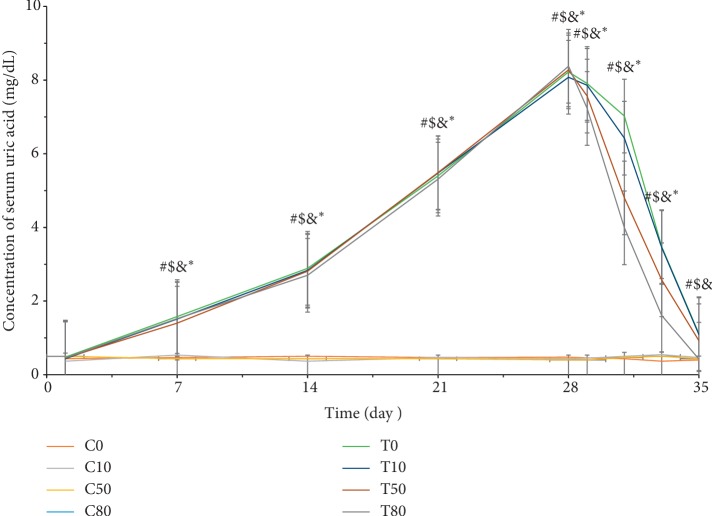
The concentration of serum uric acid changes in the treatment of hyperuricemia during OA induction and oxygenated water treatment. Data are expressed as means ± S.E.M. (*n* = 10) (^#^*p* value < 0.05 of T0 compared to C0; ^$^*p* < 0.05 of T10 compared to C10; ^&^*p* < 0.05 of T50 compared to C50; ^*∗*^*p* < 0.05 of T80 compared to C80).

Figure 5 shows the hyperuricemia in rats following 4 weeks of induction. The concentration of serum uric acid increased from 0.48 mg/dL to 8.22 mg/dL in T0, 0.43 mg/dL to 8.08 mg/dL in T10, 0.46 mg/dL to 8.28 mg/dL in T50, and 0.46 mg/dL to 8.48 mg/dL in T80. Subsequently, hyperuricemic rats were given different doses of oxygenated water for one week. As a result, a significant reduction in serum uric acid concentration was observed (i.e., 1.11 mg/dL in T0, 1.09 mg/dL in T10, 0.92 mg/dL in T50, and 0.42 mg/dL in T80).

In this study, we observed that consumption of oxygenated water could reduce the uric acid in the serum of hyperuricemic rats compared to the serum of those drinking regular water. Humans normally drink 20–30 mL of liquids, mainly plain water, per kg body weight per day. As reported previously, water absorbed by the intestines enters the enterohepatic circulation and is excreted by the kidneys. Oxygenated water consumption increases oxygen supply to the body. Oxygen has been shown to diffuse from the stomachs of young felines [9] and recent studies in rabbits indicated that intragastrically administered oxygenated water, containing more than 45 mg oxygen per liter, delivers oxygen into the abdominal cavity and the portal vein. These investigators observed an increase in pO_2_ in the abdominal cavity and the portal vein within 20–40 min. In this animal study, the increase in pO_2_ and time sequence correlated directly with the concentration of oxygen in the water [4]. According to Sommer et al., supplying oxygenated water to 3- to 6-month-old female mice for 22 weeks increased their body weight significantly for the first two weeks [10]. Weight gain may occur because of the higher oxygen concentration, because high oxygen concentrations result in enhanced rates of oxygen absorption by the body leading to increased glycolysis and mitochondrial protein synthesis [11–13]. Increased glycolysis and mitochondrial protein synthesis rates promote myofibrillar protein synthesis [14]. Therefore, drinking oxygenated water might increase oxygen concentrations in the blood and cause secondary effects. Oxygenated water might enhance cellular activity and metabolism of the liver resulting in frequent urges and urination to reduce uric acid levels in the serum. These studies may explain the change in the concentration of serum uric acid, and drinking oxygenated water could raise the clearance rate of uric acid in the plasma compared to drinking regular water. On the other hand, average daily water intake, body weight, and renal function, i.e., glomerular filtration rates, are also factors that influence serum uric acid levels. However, these parameters were not explicitly measured due to study design limitations. Further studies will be required to fully explore the relationships between these factors and uric acid levels.

## 4. Conclusions

In summary, in the current study, we observed that the concentration of serum uric acid was increased following a four-week induction with OA, and the concentration of serum uric acid decreased to a normal level after oxygenated water treatment at high doses. Therefore, oxygenated water could improve uric acid metabolism or the rate of increase in serum uric acid concentration in hyperuricemia rats compared to regular water.

## Figures and Tables

**Table 1:** List of diet controls.

Methods	Mechanisms
Increase water intake	Water is required to flush the uric acid formed in the body
Reduce intake of purine-rich food	Uric acid is formed by breakdown of purines
High fiber food	Foods high in fiber absorb uric acid present in the bloodstream, thus easily eliminating it through kidneys
Avoid intake of caffeine and alcohol	Caffeine and products containing caffeine, such as coffee, tea, carbonated drinks, and alcohol, hinder excretion of uric acid from the bloodstream by binding it
Consume right amount of vitamin C rich foods	Vitamin C helps in excretion of uric acid
Reduce sugar intake	Sugar interferes with excretion of uric acid
Consuming apple cider vinegar	Apple cider vinegar contains acetic acid which turns alkaline in body making the environment alkaline

**Table 2:** Experimental design of the animal study.

Group	Oxygenated water (mL)	Distilled water (mL)
*Control*		
Regular control (C0)	0	80
Low dosage control (C10)	10	70
Medium dosage control (C50)	50	30
High dosage control (C80)	80	0

*Prevention*		
Regular prevention (P0)	0	80
Low dosage prevention (P10)	10	70
Medium dosage prevention (P50)	50	30
High dosage prevention (P80)	80	0

*Amelioration*		
Regular amelioration (A0)	0	80
Low dosage amelioration (A10)	10	70
Medium dosage amelioration (A50)	50	30
High dosage amelioration (A80)	80	0

*Treatment*		
Regular treatment (T0)	0	80
Low dosage treatment (T10)	10	70
Medium dosage treatment (T50)	50	30
High dosage treatment (T80)	80	0

**Table 3:** Oxygen concentration of oxygenated water at different temperatures.

Temperature (°C)	Oxygen concentration (mg/L)
10	37.0
15	37.0
20	38.5
25	40.6
30	36.9
35	34.3
40	33.2
45	32.5
50	32.0

**Table 4:** Oxygen concentration of oxygenated water after different standing times.

Time (h)	Oxygen concentration (mg/L)	Time (h)	Oxygen concentration (mg/L)
1	29.2	13	17.8
2	26.4	14	17.7
3	25.0	15	17.6
4	21.9	16	17.5
5	20.1	17	17.4
6	19.2	18	17.3
7	18.9	19	17.2
8	18.6	20	17.1
9	18.4	21	17.0
10	18.2	22	16.9
11	18.0	23	16.9
12	17.9	24	16.8

## Data Availability

The datasets used and/or analyzed in the current study are available from the corresponding author on reasonable request.
